# Exploring the need and potential of ambulatory pharmacy practice for empowering patient and care delivery in India

**DOI:** 10.3389/frhs.2024.1399621

**Published:** 2024-08-06

**Authors:** Ravindra P. Choudhary, Srikanth M. Siddalingegowda

**Affiliations:** Department of Pharmacy Practice, JSS College of Pharmacy, JSS Academy of Higher Education and Research, Mysore, India

**Keywords:** clinical pharmacy, ambulatory pharmacy, patient empowerment, implementation, patient care, pharmaceutical care

## Abstract

In recent years, rapidly changing disease profile patterns, shortage & uneven utilization of healthcare professionals contributed massive burden on the Indian healthcare system, which resulted in varying, fragmented, inconsistent healthcare delivery to the patients and poor patient management. Patients often face and experience many challenges like lack of accessibility, poor patient–healthcare provider relationships, and inadequate quality of care, resulting in unnecessary economic burden in managing their health conditions. Thus Indian healthcare reform is essential in enhancing its capacity to fulfill patients' health needs that can be addressed by focusing on key sustainable strategies and initiatives meant for enhancing coordination of care, expanding services accessibility, redeveloping healthcare infrastructure, implementing workforce innovation and strong governance with the incorporation of core principles such as patient-centeredness, integrated care and collaborative care approaches. The clinical and ambulatory pharmacy practice are fragment of the healthcare delivery which delivers pharmaceutical care and fulfils the needs of patients across healthcare settings. This paper focuses on the present & future perspectives of ambulatory pharmacy practice in India and the factors to be considered for implementing it in patient care.

## Introduction

The pharmacy profession encourages pharmacists to adapt and improve their skills as they advance in practice (1). Over time, remarkable changes were observed in pharmacy practices, particularly with the introduction of clinical pharmacy and pharmaceutical care principles in the twentieth century. This broadened pharmacists' roles beyond traditional dispensing to include patient care services, allowing them to collaborate with other frontline healthcare providers (2). Whereas, pharmacists began to recognize the major issues in patient care. Polypharmacy, drug noncompliance, medication errors, and medication use safety concerns were all addressed (3, 4). These problems have surely enhanced the demand for pharmacists' clinical practice skills as well as their ability to implement a new paradigm into healthcare architecture (5).

After that, some countries saw a huge advancement in the practice of clinical pharmacy, while others were still in its infancy (6). The United States, Canada, and Australia, for example, have trained their pharmacists to be direct patient caregivers. In Europe, the United Kingdom has successfully integrated clinical pharmacy services into its practice. Northern Ireland has started an integrated medicines management (IMM) program to enhance the quality and safety of medications, improving patient health. Scotland has developed a proactive, well-structured polypharmacy management program that incorporates Quality Outcome Framework (QOF) (5).

However, in the Czech Republic, extensive educational initiatives have cleared the way for the establishment and reimbursement of clinical pharmacy services, prompted by legal and accrediting changes. Slovenia has made great advances in clinical pharmacy practice over the past decade, driven by highly qualified and motivated clinical pharmacists, as well as assistance from the national insurance company and the Ministry of Health. The adoption of Collaborative Practice Agreement-based models (CPA) from the United States has resulted in tremendous growth in clinical pharmacy throughout Saudi Arabia (7).Some countries have yet to considerably expand clinical pharmacy services. However, steps have been taken to improve education and practice, providing the framework for future clinical pharmacy service (CPS) implementation (5).

Even though clinical pharmacy services have advanced in global health systems, the process of implementing CPS is complex, difficult, and influenced by a number of variables. Gaining a thorough grasp of the obstacles that could prevent CPS from being implemented in a country's health systems is essential (8). For example, Kuwait provides a wide range of clinical services, but the absence of formal policies on pharmacy practice has impeded consistent provision among state hospitals (9). China, meanwhile, was confronted with issues such as a dearth of directives, outmoded university courses, a paucity of personnel, inadequate incentive programs, and subpar on-the-job training (10). Clinical pharmacy practice in Nigeria is still in its early stages, with several impediments highlighted, including pharmacists' lack of confidence, a paucity of pharmacy professionals, and underutilization of pharmacy technicians (11). Clinical pharmacy in Pakistan is still in its early stages, restricting pharmacists' involvement in direct patient care due to a lack of clinical components in their curriculum (6). Vietnam has achieved some progress in clinical pharmacy activities, but there is still need for improvement in patient-specific activities (12).

Navigating implementation challenges can be confusing because they change based on the context. For example, in Brazil, CPS have been integrated into the Brazilian Health System (SUS) throughout the last decade. However, problems such as insufficient physical infrastructure in health centers and ineffective marketing techniques have hampered widespread implementation (8). In Tanzania, efforts have been made to change pharmacy rules to incorporate CPS as a key function for pharmacists. Additionally, a sponsorship scheme has been launched to boost the number of clinical pharmacists in the country. However, the availability of CPS in Tanzania remains minimal (13). Furthermore, acceptance of identical clinical pharmacy services differs by healthcare provider. This disparity can be related to the diverse roles of clinical pharmacists (CPs) across countries. For example, in Slovenia, CPs do not have prescription privileges, although in the United States, such rights could boost acceptance rates and collaboration (14).

Despite various hurdles, clinical pharmacists play an important role in enhancing therapeutic, safety, and humanistic outcomes by providing unique insights and valuable suggestions on safe pharmaceutical usage and patient care. Furthermore, their experience not only improves patient care, but it also helps to reduce costs and mitigate treatment-related risks. Thus, integrating clinical pharmacy services is critical to improving patient care and safety (15–17). It is clear that countries that have implemented CPS are reaping a variety of benefits, including improved drug utilization, drug safety, and medication adherence. These developments are critical for ensuring the viability of respective healthcare systems (5).

Clinical pharmacy practice has a history of promoting healthcare through innovation (18). As novel practices have been adopted, several new areas of clinical pharmacy practice have emerged. One such specialty is ambulatory care pharmacy. This specialized field is rapidly expanding, as clinical pharmacists collaborate with healthcare teams to guarantee the safe and effective administration of pharmaceuticals (19). Ambulatory care pharmacy practice involves pharmacists providing essential healthcare services to meet patients’ medication needs through strong patient partnerships and a community-oriented approach. They provide direct treatment, develop long-term patient connections, collaborate with other healthcare practitioners, advocate for patients, promote wellness, and educate on self-management. Ambulatory pharmacists work in a variety of settings, including community and institutional clinics, primary care, specialty care, and telehealth, and serve a diverse population of patients (20). Creating new ambulatory pharmacy services for the community might be intimidating (21). It entails addressing critical issues such as pharmacist training and competency, physical needs, service structure, and successful implementation strategies (7).

### Patient experience with existing healthcare: current scenario

India has a population of 1.40 billion inhabitants (22). The country is undergoing a demographic shift, with an expanding geriatric population. This, together with the increasing burden of non-communicable diseases such as cardiovascular disease, cancer, diabetes, mental health issues, and injuries, places enormous demand on healthcare systems (23, 24). The country has both governmental and private healthcare facilities to mitigate the impact of disease burden. However, most private healthcare providers provide service at varying levels in metropolitan areas (25). Approximately 80% of the population prefers private health institutions for outpatient care due to their perceived superior quality. Lower-income people, on the other hand, prefer to use public health facilities since they provide affordable care. The underutilization of basic healthcare services in public institutions is mostly attributable to ineffective response. Furthermore, the differing cost and quality of private facilities cause disparities in affordability and healthcare experiences based on socioeconomic status (26).

Over the last two decades, the country's inpatient and outpatient load has risen dramatically. The nation's health workforce totals around 5.7 million people, although there is a general shortage of healthcare professionals due to their uneven distribution among states. As a result, many healthcare practitioners across the country engaged in dual practice to handle the patient load, raising worries about prescription errors and drug-related issues (23). Dual practice is a prevalent practice among healthcare practitioners globally. In the United Kingdom and Northern Ireland, more than 60% of public hospital doctors also practice privately. Similarly, over 80% of public-sector physicians in Egypt, Indonesia, Kenya, and Mexico engage in private practice (27). Physicians in low- and middle-income nations such as India, Egypt, and Vietnams frequently practice dual roles (28).

Dual practice, when properly regulated, has the potential to improve access to healthcare services while also diversifying the treatments accessible to patients. However, inadequate regulation can have a negative influence on public health service access, quality, efficiency, and equity (27). As a result, several developed countries have enacted restrictions governing dual practice. For example, in France, public physicians' revenue from private patients cannot exceed 30% of their overall pay. Full-time NHS consultants in the United Kingdom can earn no more than 10% of their NHS pay from private practice. In contrast, countries such as the United States and Canada have outright banned this practice. However, India lacks such rigorous regulations on dual practice (28).

Inappropriate prescriptions are frequently found among practitioners of both traditional and non-traditional healthcare systems who prescribe allopathic drugs. Furthermore, healthcare workers functioning in the allopathic medicine system frequently meet cases of incorrect prescribing (29). Furthermore, there are hints of unreliable link between prescribers' and dispensers' of medications, with unnecessary prescriptions. Additionally, innumerable unqualified healthcare offer healthcare services across the nation and medications are often dispensed by informally trained staff in pharmacies, leading to irrational use of drugs. These circumstances lead to long-term treatment complications, unnecessary hospitalizations, and readmissions, burdening patients both emotionally and financially (23, 30).

The widespread fragmentation of the healthcare industry has led to the management of patients' clinical issues by several different healthcare providers, which frequently results in extremely disorganized, inefficient, and lengthy patient pathways. This fragmentation has a negative impact on the continuum of care, the quality of care, and the rationality of treatment plans. As a result, it leads to poor illness management, illogical pharmaceutical use, greater risk of medication-related problems, a higher incidence of adverse events, more expensive interventions, and higher out-of-pocket healthcare costs. These problems prohibit the best clinical, economic, and humanistic outcomes for patients requiring long-term care. In India, most health insurance programs only cover hospitalization costs and not outpatient care costs, and they have inadequate referral links, resulting in overpopulation in secondary and tertiary health institutions (23). As a result, continual and concurrent monitoring, review, and study of health systems is critical for tracking changes and making essential modifications (31).

### Need of ambulatory pharmacy practice in patient care

Health sector reform addresses issues of equity, efficiency, quality, finance, and sustainability in healthcare. This includes establishing priorities, improving policies, and altering the organizations in charge of policy execution (31). Currently, health systems are focused on attaining the quadruple goal of improving patient experience, improving population health, lowering costs, and fostering a better work-life balance among healthcare personnel. Clinical pharmacists are well-positioned to be key stakeholders in this dynamic context, facilitating successful collaboration to achieve the quadruple aim (32). By actively expanding ambulatory care pharmacy practices, the pharmacy sector will make a substantial contribution to national issues such as improving patient care, public health, and making healthcare more affordable (33). Ambulatory pharmacy practice is an essential component of the healthcare system, providing pharmaceutical care to outpatients. Ambulatory care pharmacists are responsible for increasing patients’ understanding of correct drug usage, assessing patients' pharmaceutical needs, managing medication-related difficulties, and developing relationships with patients and their families (34).

Aging populations bring new challenges, such as multimorbidity, polypharmacy, and the engagement of several healthcare professionals, which complicate outpatient management. For example, it is obvious that polypharmacy contributes to the notable incidence of medication errors, adverse drug events, and other medication safety issues among patients. It is estimated that around 1.5 million preventable adverse drug events occur annually because of medication errors, at a cost of billions of dollars, and around 20% of patients had at least one medication discrepancy on admission, which could cause moderate to severe harm (3, 35). In India, almost 50% of families spend on unnecessary healthcare investigations and prescriptions. Adverse drug reactions are indicators of prescription misuse caused by poor prescribing practices and a lack of compliance (36). Despite rising demand for quality healthcare, India's healthcare delivery remains inadequate, owing to the multiple problems associated with patient care (37).

### Challenges of medication errors

Medication errors (MEs) are a major public health concern, compromising patient safety, undermining trust in the healthcare system, raising expenses, and lowering quality of life (38). In India, roughly 5.2 million medication errors occur each year, with an incidence rate of 1.5 MEs per 100 prescriptions. While the majority of medication errors do not harm patients, some cause temporary or permanent ailments and, in rare circumstances, death (39). The frequency of MEs in developing nations like India remains concerning necessitating immediate action to safeguard healthcare consumers. In 2017, India ranked 143 out of 184 nations in terms of global health-related sustainable development goals. This low ranking is mostly the result of inadequate healthcare infrastructure, a lack of medical insurance coverage, high out-of-pocket expenses, and prescription errors, such as adverse drug reactions (ADRs) and MEs. Addressing these issues is critical for improving healthcare results and patient safety.

The most typical type of ME seen will be a prescription error (transcription error), followed by a dispensing or administration error. Prescription errors were primarily the result of incomplete and illegible prescriptions. For example, a study conducted in India found that the most common factors to pharmaceutical errors were a lack of time and an increased workload, with prescription errors occurring at a rate of almost 40%. This conclusion is consistent with a UK-based study that revealed a 36% rate of prescription errors. Furthermore, it is evident that numerous medication errors continue to get unreported (38, 39).

### Challenges of adverse events in treatment

Adverse events in medical treatment (AEMT) are a major global issue that are frequently caused by medical mismanagement rather than patients' underlying diseases. Unfortunately, this problem is prevalent, with around one in every ten patients being hurt while receiving care. In the United States, AEMT is the third greatest cause of mortality, and in low- and middle-income nations, it accounts for one-third of all deaths (40). The evidence suggests that the majority of adverse occurrences in medical treatment are preventable (41).

According to research, roughly 20%–25% of the general public suffers injury in primary and outpatient care settings in both developing and developed countries (42). Every year in low- to middle-income nations, safety breaches cause an estimated 134 million adverse events and 2.4 million deaths. In India, AEMT-related deaths and disability-adjusted life years (DALY) were higher than the norm for low-middle socio-demographic index (SDI) countries (43). While extensive efforts have been made to prevent AEMT-related fatalities, the number of these incidents in the UK has remained unchanged (41).

### Challenges of antimicrobial resistance

Antimicrobial resistance is a major public health concern, fueled by antibiotic overuse throughout the care continuum. An estimated 80%–90% of all human antibiotic use occurs in the ambulatory context, with evidence suggesting that 30%–52% of outpatient antibiotic prescriptions are unnecessary. Excessive antibiotic use is a leading cause of resistance and adverse effects (44). Although India is the world's largest consumer of antibiotics by volume, it lacks a structured system of antibiotic use surveillance that could guide an antimicrobial stewardship program comparable to those in the United States and Europe (45).

For instance, a study in India found that healthcare practitioners in primary care settings gave antibiotics to half of their patients for diseases that did not require them, with only a few “Reserve” category drugs being utilized. Inappropriate antibiotic usage was particularly widespread in rural areas among qualified physicians and patients with presumptive tuberculosis, resulting in significant diagnostic delays, drug-related adverse events, and increased out-of-pocket costs. It emphasizes the important need for antibiotic stewardship in outpatient settings (46).

### Challenges of medication misuse and misconceptions

Over-the-counter (OTC) or non-prescription medications are thought to be safe and effective, and they are widely available to the general population without a doctor's prescription. There are over 300,000 OTC medicinal items on the market, and the number is growing as more pharmaceuticals transition from prescription to over-the-counter status (47). OTC drugs play an important role in promoting self-care by giving simple remedies for a wide range of common health conditions. However, excessive and inappropriate use of these drugs has prompted healthcare experts to express concerns about patient safety. These concerns have grown rapidly as a result of a lack of knowledge concerning adverse consequences such as antibiotic resistance, skin issues, hypersensitivity, and allergies. Notably, many teenagers and adolescents purchase and utilize over-the-counter medications without reading the directions. Evidence suggests that individuals frequently share drugs, utilize outdated products, double doses without consulting, store them poorly, and disregard label expiry dates (48).

Topical steroids are the most commonly utilized over-the-counter medications in dermatology practice (47). However, a disturbing trend of steroid abuse among patients, pharmacists, and physicians has been widely reported in numerous research. Improper use of these drugs can result in serious local and systemic effects (49). Social media influence, peer pressure, and unethical marketing have all contributed to the abuse of topical corticosteroids, notably in fairness creams. This usage has resulted in major problems (50). On the other hand, some patients avoid corticosteroids out of fear of reported side effects, resulting in poor prescription adherence (51). Nonadherence can have an impact on disease control and put a load on healthcare resources (52). Misinformation, ignorance, and improper counseling are contributing to the rapid growth of steroid phobia. It is essential for various stakeholders, including healthcare professionals, the pharmaceutical industry, the media, and the general public, to work together to address this concerning trend and its negative impact (53).

### Challenges of medication non-compliance

Non-communicable diseases (NCDs) are a prominent cause of death worldwide, particularly in low- and middle-income nations. Treating NCDs frequently necessitates long-term use of medications (54). Adherence to pharmaceutical therapy is critical, particularly when managing chronic diseases. However, approximately half of patients do not take their drugs as prescribed (3, 54). Non-adherence greatly leads to inadequate drug responses, early hospitalizations, frequent hospital visits, and Adverse Drug Events (ADEs), all of which lead to poor treatment results and higher healthcare expenses (55). In India, poor medication adherence among NCD patients is widespread due to causes such as missed doses, discontinuation of treatment, and inability to commence medication. Nonadherence is a significant healthcare and economic burden (54).

Although software, mobile apps, patient tools, and diaries have helped to increase adherence, the problem still exists. The intricacy of prescription regimens and multiple pharmacological therapies contributes significantly to poor adherence. Complex treatment schedules, multiple daily dosages, and failure to follow precise prescription instructions (time and frequency) all have a substantial impact on adherence among patients with chronic diseases (55). Furthermore, the silent progression of NCDs, combined with insufficient awareness about these diseases, results in patient nonadherence, limiting drug use to when symptoms appear (56).

### Ambulatory pharmacy practice activities in enhancing patient experience and health outcomes

#### Comprehensive medication review: addresses medication safety related issues

Misuse of high-risk drugs can result in serious damage or even death (57). Adverse drug events and medication errors occur predominantly during care transitions and across the care continuum and are often caused by a lack of communication, medication discrepancies, poor patient education, and a lack of follow-ups (58, 59). Clinical pharmacists are responsible for conducting detailed patient interviews that include medical history, social and family history, allergy history, usage of over-the-counter medications, nutritional supplements, and alternative therapies. They examine medication therapy, using clinical and laboratory data to discover and address issues such as therapy duplication, drug-drug and drug-food interactions, contraindications, and incorrect dosages (60). Clinical pharmacists play an important role in recognizing and preventing prescription errors ensuring safe and effective clinical outcomes through medication audits and training healthcare members (39).

#### Medication reconciliation: addresses medication discrepancies

Inaccurate medication histories can result in therapy discontinuation, restart of discontinued medications, incorrect therapies, and missed drug-related issues. Up to 27% of hospital prescribing errors are the result of incomplete or erroneous drug histories at admission (61). Medication reconciliation, a vital procedure in both inpatient and outpatient settings, involves numerous healthcare providers and helps to reduce or eliminate the risk of medication errors during transitions by maintaining each patient's regimen accurate and up-to-date (62). It aids in the detection and resolution of medication discrepancies before they lead to costly and disastrous consequences.

Pharmacists are uniquely qualified to deliver patient-centered medication care, including medication reconciliation. Medication reconciliation should be done when patients switch between healthcare settings or when a drug is modified or discontinued. Several studies have indicated that pharmacists have a positive impact when integrated into the drug reconciliation process. As medication experts, pharmacists have a considerable impact on patient outcomes, especially during transitions between healthcare facilities (63).

#### Patient education: addresses health illiteracy

Health awareness is a critical component in disease prevention (56). In India, the general public is becoming more aware of the quality of healthcare services and expects better care. However, the current system is overburdened and incapable of meeting these demands. Consumer forums commonly address issues such as the inappropriate use of drugs. As the number of people seeking medical attention grows, healthcare providers find it increasingly difficult to educate patients about lifestyle changes, correct prescription usage, and illness management.

Pharmacists have multiple chances to help physicians in a variety of settings (64). Clinical pharmacists, for example, can step in when busy practitioners don't have time to explain the possible risks of long-term unsupervised medication use. Working directly with patients at the dispensing level makes it possible to predict and prevent medication misuse and abuse. This alleviates the strain on clinicians, allowing them to concentrate on patient care (50). Pharmacists play an important role in reducing self-medication by consulting and educating patients (65).

### Medication adherence support: addresses medication non-compliance

Patient behaviour, treatment regimens, and interactions with healthcare providers all have an impact on drug compliance. As a consequence, many patients do not fully benefit from therapy, leading to morbidity, mortality, and social expenses (66). Pharmacists are uniquely positioned to educate and counsel patients, boosting adherence through a variety of approaches. Clinical pharmacists can enhance medication adherence and health outcomes for patients with chronic diseases by increasing patients' understanding of their disease, promoting dietary and lifestyle changes, and ensuring optimal medication usage (67). Furthermore, pharmacists provide new medicine services to newly diagnosed chronic disease patients. This comprises of three steps: offering initial guidance and information while administering the medicine, performing a two-week follow-up to ensure adherence and address obstacles, and holding subsequent discussions to review progress and answer additional inquiries (57).

### Ambulatory antibiotic stewardship: addresses antimicrobial resistance

The high prevalence of communicable diseases and excessive antibiotic usage in Indian healthcare settings demands the immediate implementation of comprehensive antimicrobial stewardship programs (ASPs) (68). To meet the growing demand for antibiotic stewardship in outpatient settings, pharmacists play a critical role in developing effective pharmaceutical treatments and guiding innovative stewardship models (69). There is evidence that antimicrobial stewardship initiatives in ambulatory settings improve antibiotic medication choices and prevent inappropriate use. Furthermore, studies have demonstrated that pharmacist-led antimicrobial stewardship initiatives reduce overall antibiotic use, costs, and therapy duration in a variety of healthcare settings (70).

Integrated rapid diagnostic methods and real-time ASP interventions have been considered as potential solutions for developing clinical pharmacy services (71). Furthermore, the growth of pharmacist positions has been associated with increased stewardship actions and better clinical outcomes. The CDC's Core Elements of Antimicrobial Stewardship and the Quality Innovation Network-Quality Improvement Organization (QIN-QIO) Field Guide provide valuable assistance for developing effective stewardship programs in outpatient care (44).

### Patient-centered care communication: addresses healthcare users’ needs

Pharmacists evaluate drug problems, determine causes, and recommend corrective steps, prioritizing issues based on patient needs and preferences (57). Vulnerable adults at home frequently suffer MRPs because of polypharmacy and severe health problems (72, 73). Medication reviews are an organized evaluation of medications to improve treatment to produce the optimum outcome (72). Medication reviews have been recognized as critical to addressing inappropriate polypharmacy (74). Domiciliary medication reviews (DMRs) are described as in-depth, complete medication reviews focused on the individual's needs, and they have grown more common in recent years. A domiciliary pharmacist provides medication optimization services and, when applicable, ensures a smooth transition of care between various healthcare venues and the patient's home (73).

A complete DMR eliminated confusion and worry around the appropriateness of pharmacological therapy. As a result, informal caregivers respected professional knowledge and felt confident that someone had taken the time to verify medications used were appropriate for their family member (74). For example, the Australian Home Medicines review (HMR) program allows pharmacists to examine patients' medications at home and report findings to their GP in order to optimize drug management (75).

### Public health: aiding health promotion and preventive care

Pharmacists are encouraged to work with other healthcare professionals to actively provide public health services that promote wellness and population health. They are responsible for providing screening and monitoring services. Screening services involve validated instruments to collect information about risk factors and may include point-of-care testing to determine the nature and amount of risk. When combined with education, guidance, and referrals, these activities promote early detection and comprehensive assessment. The monitoring service additionally ensures that chronic condition patients adhere to medicine and non-pharmacological therapies, as well as identify disease progression (57).

Accurate health and drug information is critical due to the abundance of a variety of sources, some credible and others not, which can lead to misconceptions and unhealthy lifestyle choices. The healthcare system requires evidence-based, properly conveyed, and consistent drug information (57). Pharmacists are significant resources because of their availability and competence in health and drug knowledge. Their important location in the community, as well as their clinical competence, make them critical, as their activities are intimately related to critical health services for public health (73). Medicines information services serve both the general public and healthcare professionals by assuring safe medication use and improving patient care (57).

### Key strategies in implementing ambulatory pharmacy practice in India

#### Workforce allocation, education, and training

In India, the Pharm.D program, a postgraduate doctoral course, was established in 2008 to train clinical pharmacists who are capable of working with other healthcare providers to fulfill patient needs. However, it has been found that Pharm.D graduates who work as clinical pharmacists do not have access to additional training programs to improve their professional abilities, perhaps contributing to a lack of expertise in their practice (76). In contrast, several European countries demand particular specializations or continuing professional development training for employment in the clinical pharmacy sector (77). A study found that Indian Pharm.D students perceive a significant volume of theory-based instruction that does not correspond with practical training, and similar findings have been documented in European studies (76, 77).

Several factors contribute to poor professional competency, including a shortage of trained faculty, preceptors, and practical training opportunities. These concerns highlight the importance of specialization and continuing professional training, as well as the need for experienced faculties and preceptors, along with an updated curriculum, to foster professional competency of clinical pharmacists in the country. Furthermore, clinical pharmacists and other healthcare professionals have taken advantage of social media channels to demonstrate their professional competence. The pharmacy council should establish a uniform forum where healthcare professionals can discuss the most recent achievements in their disciplines and address associated challenges (76).

#### Restructuring practice sites

The selection and evaluation of the practice place is far more complicated. Physical requirements such as accessibility, workspace, and necessary equipment must be considered when developing an ambulatory pharmacy practice. Documentation storage facilities, as well as discussion and presentation spaces, have been constructed to extract useful material for interactive use during interprofessional education (21). Information technology is critical to optimal patient care delivery, outcome analysis, and assessment (78). Implementing and using electronic health records (EHRs) is one of the keys to a successful ambulatory care practice (79).

#### Collaboration policies

According to World Health Organization experts, interprofessional care should be a standard practice in patient care because it is critical to providing high-quality health care. Pharmaceutical care is an example of a patient-centered interprofessional service that requires collaboration between physicians and pharmacists (80). This collaboration is often formalized through a Collaborative Practice Agreement (CPA), in which a licensed healthcare provider supervises patient care and refers patients to the pharmacist. This protocol allows pharmacists to perform patient-specific care functions such as patient assessments, counseling, referrals, ordering laboratory tests, administering drugs, and managing drug therapy regimens (81).

In many countries, like the United States, physicians and pharmacists collaborate formally through CPAs (80). These collaborative relationships are based on trustworthiness, role specification, and professional contacts. Furthermore, the United States government recognized pharmacists as healthcare providers for Medicare beneficiaries. The toolkit created by the Centers for Disease Control and Prevention (CDC), the National Alliance of State Pharmacy Associations (NASPA), and ChangeLab Solutions served as a resource guide for creating CPAs. These measures have considerably accelerated the pharmacy profession's incorporation into healthcare teams (82).

#### Regular services documentation and quality check evaluation

Proper documentation of ambulatory care pharmacy services is critical because it acts as a valuable communication tool for healthcare professionals. Documentation can serve as supporting evidence for research and instructional purposes. It emphasizes the importance of the clinical pharmacist's accountability and services. Furthermore, appropriate documentation might improve the chances of compensation. Documentation serves as evidence in legal actions and can also be used to measure service quality, consequently improving ambulatory practice (83).

#### Advancing towards reimbursement model development

The development of effective clinical pharmacy services is dependent on the establishment of successful reimbursement mechanisms (5). Integrating new patient care services into business models necessitates utilizing existing medical payment structures, which have traditionally excluded pharmacists due to their lack of provider status (84). However, in the United States, clinical pharmacists have achieved provider status, allowing them to be reimbursed for their services, but the extent varies (85).

In Europe, key instances include the Czech Republic and Slovenia, which successfully adopted funded clinical pharmaceutical services. The Czech Republic's legislation defined clinical-pharmaceutical care, distinguishing clinical pharmacists' specialized function from typical pharmacy services, resulting in rapid progress in further legislation and funding schemes for acute and ambulatory care. Similarly, Slovenia's updated pharmacy Act, which clearly defines clinical pharmacy services, paved the door for fully paid services like medication reconciliation and seamless care (5, 86). Germany has also received payment for five clinical pharmaceutical services after two decades of extensive research and negotiations (87).

These examples emphasize the value of clinical pharmacists' active participation in reimbursement negotiations, health policy creation, and public health authorities. National policies and regulations must be aligned to support clinical pharmacy programs, necessitating a “top-down” approach. Developing nations can establishment of new clinical pharmacy services, but needs appropriate laws and the creation of frameworks that draw on the experiences of other countries (5). However, issues remain in scaling up and maintaining the long-term availability of these services. Implementation research is critical for improving pharmaceutical care programs and effectively addressing these challenges (87).

## Conclusion

Ambulatory care pharmacy practice is a growing, promising, reward-worthy patient care delivery and practice initiative that focuses on enhancing patients' health outcomes and fulfilling pharmaceutical care. Ambulatory pharmacy practice services will eliminate gaps present in the current care delivery approaches & reduce the burden on the healthcare system. It also helps in the advancement of the clinical pharmacy profession in India and provides an opportunity for budding clinical pharmacists to practice. So Indian healthcare directorates must consider and strategic plans must be executed for effective implementation of these initiatives based services to meet the health needs of the population.
